# Musculoskeletal disorders and symptom severity among Australian dental hygienists

**DOI:** 10.1186/1756-0500-6-250

**Published:** 2013-07-04

**Authors:** Melanie J Hayes, Derek R Smith, Jane A Taylor

**Affiliations:** 1 School of Health Sciences, Faculty of Health and Medicine, University of Newcastle, PO Box 127, Ourimbah, 2258, Australia

**Keywords:** Musculoskeletal disorders, Dental hygienist, Occupational health

## Abstract

**Background:**

Recent literature has identified that musculoskeletal disorders (MSD) are a significant occupational health issue for both dentists and dental hygienists. Research on the occupational health of dental hygienists is lacking in Australia, which is of particular concern given that it is a rapidly growing field in this country. The aims of this research are to investigate the prevalence of MSD and correlating regions of pain among Australian dental hygienists. A self-reporting questionnaire was distributed to all registered dental hygienists in Australia. The questionnaire was a modified version of a validated tool, used previously among health practitioners and students.

**Results:**

A total of 624 dental hygienists responded to the questionnaire, achieving a response rate of 42%. MSD were frequently reported by dental hygienists in the neck (85%), shoulder (70%), and lower back (68%). Of those reporting pain, over two thirds reported that the pain lasted for longer than two days, for all body regions. Logistic regression analysis revealed that there is a correlation between reports of MSD in the neck, shoulder and lower back regions.

**Conclusions:**

Overall, this study suggests that MSD are a reasonably common problem for Australian dental hygienists, and that they often need to seek medical treatment for these problems. It is concerning that there is a correlation between reports of MSD in the neck, shoulder and lower back regions; further studies are needed to establish the epidemiological patterns of MSD in this profession.

## Background

Musculoskeletal disorders (MSD) are a costly health problem, with an estimated 4.6 billion dollars being spent on these conditions in Australia annually [1]. Work-related MSD is a major concern for dental practitioners, where daily work tasks sometimes place them at a high risk of injury [2]. Dentistry is visually demanding, and requires the adoption of fixed postures and the use of repetitive and precise hand and finger movements. It is considered that these biomechanical risk factors combined with psychosocial stress contribute to the development and progression of MSD [3,4]. Despite this knowledge, MSD prevalence rates continue to be high amongst dental professionals, especially among dentists and dental hygienists.

Previous investigations in Australia have found that dentists suffer MSD at reasonably high rates, with 64% of New South Wales (NSW) dentists in one study reporting MSD in the previous month [5], while over 87% of Queensland dentists reported pain in the past 12 months [6]. Similarly high rates have also been reported by dentists in Thailand (78%) [7], Sweden (78%) [8] and Lithuania (91%) [9]. A recent systematic review revealed back pain as the most commonly reported MSD among dentists, while reports of neck pain might also be high, but have been less consistent across studies [10]. Conversely, dental hygienists appear to more commonly report hand/wrist pain, with prevalence rates of 64% and 69% being reported in Sweden [8] and the USA [11]. Dental and dental hygiene student cohorts have also reported MSD at similar rates to the profession, with over 60% of those surveyed reporting neck pain in Australia [12,13].

In the last decade, a number of new training programs have emerged in dental hygiene in Australia. As such, many dental practices have expanded their dental team to include a dental hygienist. While studies in the United States and Europe have identified MSD as a common complaint of dental hygienists, very little is known about the prevalence of MSD among Australian dental hygienists.

The aim of this study, therefore, was to investigate the prevalence of MSD and symptom severity among Australian dental hygienists.

## Methods

This project was approved by the University of Newcastle Human Research and Ethics Committee. All registered dental hygienists in Australia were contacted via a single mail-out using State/Territory Dental Board listings in 2010. Each registered hygienist received a participant information statement outlining the research project, a five page survey relating to work habits and MSD and a reply-paid, return-addressed envelope. The self-reporting survey was comprised of 54 questions, and was a modified version of the Standardized Nordic questionnaire (SNQ) [14]. The SNQ is a reliable and valid tool that has been used repeatedly among health professionals and students in various countries, to investigate MSD [15-20]. Respondents were asked to provide information regarding social habits, qualifications and education, work habits and musculoskeletal symptoms. With the aid of a diagram, the body was divided into 11 identifiable regions, and for each region respondents were asked whether they had experienced any MSD in the previous 12 month period, and whether the pain had lasted more than two days, affected their daily life, or required medical attention. Consent was implied by the return of a completed anonymous survey. Data was entered into a spreadsheet and analyzed using statistical software to determine prevalence rates, while logistic regression analysis was performed to determine correlating regions of pain.

## Results

### Respondent profile

A total of 624 questionnaires were returned, which represented 42% of all surveys distributed. A small selection of surveys were excluded from the analysis (n = 64), due to the respondents not primarily working as a dental hygienist, or incompleteness; as such, a total of 560 surveys were included in the final analysis. The profile of respondents was consistent with National Dental Labour Force data [21]; that being predominantly female (96.1%), with a mean age of 36.5 years. Only a small percentage (5.4%) identified themselves as regular tobacco smokers, while half (56.6%) indicated that they regularly consumed alcohol. The respondent profile suggests many hygienists work part-time (averaging 3.6 days per week) in general private practices (77.1%), with an average of 45 minutes scheduled for each patient appointment. The characteristics of respondents are summarized in Table 1.

**Table 1:** Characteristics of dental hygienists

	**Mean**	**SD**	**%**
Age (years)	36.5	9.5	
Height (cm)	165.7	9.0	
Weight (kg)	65.6	14.3	
Years working as a dental hygienist	11.8	9.7	
Days working per week	3.6	1.2	
Hours per week			
<8			7.3
9-16			10.2
17-24			16.8
25-32			26.9
>32			38.7
Patients per day	11.3	9.0	
Appointment time per patient (minutes)	45.4	12.4	
Type of practice*			
General Private			77.1
Orthodontics			19.2
General Public			13.3
Periodontics			7.2
Paedodontics			2.0
Other			2.2

*May total >100%, as some respondents work at multiple practices.

### Musculoskeletal disorders

**Figure 1:**
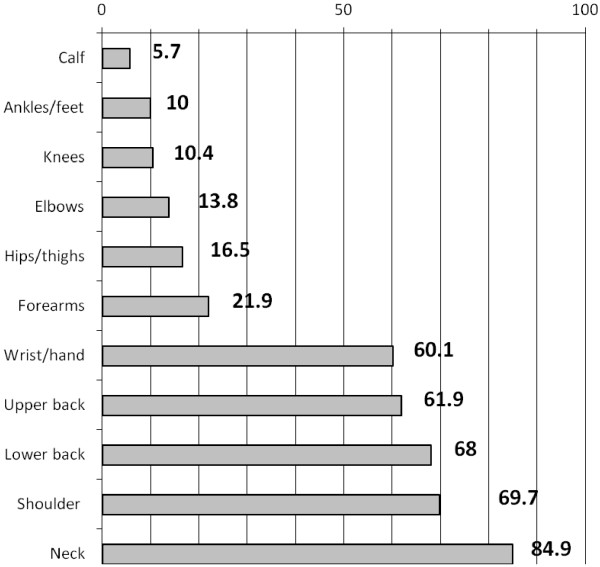
12 month prevalence of MSD reported by dental hygienists, by body region (%).

The prevalence of MSD, by body region, is summarized in Figure 1. More than two-thirds of respondents indicated that they had experienced MSD in the neck, shoulder and lower back regions in the past 12 months, while more than half had experienced upper back and wrist/hand MSD. MSD in the lower extremities (hips/thighs, knees, calf/lower leg, ankles/feet), was reported by less than 17% of the respondents.

### Symptom severity

The number and proportion of respondents who indicated that their MSD lasted longer than two days, affected their daily life, or required medical attention is presented in Table 2. More than two-thirds of those reporting MSD indicated that their pain lasted more than two days. In nearly all body regions (the exception being forearms), half of those reporting MSD revealed that their pain affected their daily life. Those hygienists who required medical treatment as a result of their MSD represented between 36% (wrist/hand) to 67% (hips/thighs) of those experiencing any MSD.

**Table 2:** Symptom severity among Australian dental hygienists reporting MSD

	N*	**% reporting MSD lasting >2 days**^**# **^**(n)**		**% with symptoms affecting daily life**^**# **^**(n)**		**% required medical treatment**^**# **^**(n)**	
Neck	473	74.6 (353)		60.7 (287)		56.5 (267)	
Shoulders	388	73.5 (285)		57.7 (224)		53.6 (208)	
Upper back	345	75.4 (260)		55.1 (190)		57.1 (197)	
Elbows	77	77.9 (60)		52.0 (40)		45.4 (35)	
Forearms	122	68.0 (83)		48.4 (59)		47.5 (58)	
Wrists / hands	334	64.5 (216)		50.1 (167)		36.1 (121)	
Lower back	378	69.7 (264)		58.9 (207)		52.0 (197)	
Hips / thighs	92	78.3 (72)		57.6 (53)		67.4 (62)	
Knees	58	77.6 (45)		56.9 (33)		44.9 (26)	
Calf / lower leg	32	81.3 (26)		62.5 (20)		59.5 (19)	
Ankles / feet	56	80.3 (45)		50.0 (28)		41.0 (23)	

*N = number of respondents reporting any musculoskeletal symptoms in that body region.

^#^ % presented as a proportion of *N*.

### Correlations

Dental hygienists experiencing neck MSD are more likely to report MSD in the shoulder (OR: 6.88, 95%CI 4.10-11.67, p<0.01) and lower back (OR:2.96, 95%CI 1.77-4.95, p<0.01). Shoulder MSD also statistically correlated with lower back MSD (OR: 1.63, 95%CI 1.08-2.47, p<0.02).

## Discussion

This study explored the prevalence of MSD and symptom severity among a complete cross-section of Australian dental hygienists. Their 12 month prevalence of neck MSD was similar to a previous study of Australian dentists [6], and studies of hygienists in other countries including the United States [22] and Canada [23]. While many Australian dentists have reported MSD in the shoulders, wrists/hands and lower back, in the present study hygienists reported upper back MSD more frequently than dentists [6]. This is an interesting finding, as previous research has indicated that hygienists are more likely to report hand/wrist MSD [10]; this is thought to have been due to repetitive scaling tasks performed daily as a hygienist. However, a recent study has identified that it is not only work-related tasks that are risk factors for MSD among dental hygienists, but also psychosocial factors, such as a lack of involvement in practice decisions and work interference in home life [24].

It is noteworthy that of the hygienists who reported MSD, more than two-thirds reported the pain lasting longer than two days, for each body region. Interestingly, a study of Australian dentists found that female dentists reported more frequent MSD symptoms (particularly pain and headaches) than their male counterparts [5]. Similarly, female dentists in Brazil and Lithuania reported a higher incidence of MSD compared with males [9,25]. It is concerning that hygienists may be ‘working through the pain’ and potentially exacerbating any injury. Studies have also shown that female dentists rate their MSD symptoms worse than male dentists [5,7]; this may be concerning as the dental hygiene profession is predominantly female [26]. The reason for the notable difference in how females report MSD is unclear, although it has been suggested that females may pay more attention to their health and well-being, or that they may have a lower pain threshold or are less resistant to constant musculoskeletal tension [9]. Furthermore, the fact that neck, shoulder and lower back MSD were inter-correlated in the current study indicates that dental hygienists are suffering from MSD in more than one body region. Whether the MSD is occurring concurrently cannot be elucidated from this study; however, it is evident that a significant proportion of the workforce is suffering from MSD, and that it is interfering with their daily life.

It is also concerning that many hygienists reported the need to seek medical attention for their MSD. Over one-third sought medical advice or treatment for MSD in the neck, shoulders, upper back and lower back; these reports are similar to Australian and Saudi Arabian dentists, where over one-third had sought medical attention for MSD [6,27]. It has been suggested that an injured worker often bears significant financial and social costs coupled with their MSD [28], these factors in combination may contribute to dental hygienists taking sick leave, reducing their hours or even leaving the profession. A survey of dental hygienists in the United States found that those with pre-existing pain were absent from practice due to MSD for an average of five weeks per year [29], while more than 40% of dentists in the United States reduced their work hours due to MSD [30] and Swedish dentists with a high prevalence of MSD were more likely to leave their profession [8]. Appointments to visit doctors or physiotherapists, while beneficial and necessary for MSD, may impact negatively on dental hygienists financially in the form of increased medical costs or lost wages, the dentist-employer by way of lost income due to decreased practice productivity, and even patient’s health and finances due to the rescheduling of appointments. This wider impact demonstrates the need for determining protective factors or interventions to limit the prevalence of MSD in the profession. It should also be noted that studies have shown that premature ill-health retirement is most commonly attributed to MSD among dentists [31].

The present study required participants to complete a self-reporting survey, which may conceivably introduce some level of response bias and limit the generalizability of results. Nevertheless, this bias was limited as much as possible by utilizing a survey proven to be a valid and reliable tool for measuring the prevalence of MSD [32]. Another potential limitation might relate to the calculation of response rates, as the bulk of surveys were posted by State/Territory Dental Boards. This meant that the researchers did not have direct access to databases to track the number of surveys actually sent out or “returned to sender”; a limitation which also limited the follow-up of non-respondents. Even so, we did utilize many other methods proven to increase response rates in survey-based studies, including keeping the questionnaire as short as possible, the use of stamped return envelopes, and using the University logo on all stationary [33]. The demographics of respondents in the present study were consistent with National Dental Labourforce data, and so it may be assumed the respondents were fairly representative of the profession as a whole.

## Conclusions

Overall, our study suggests that MSD are a reasonably common problem among Australian dental hygienists, with many reporting that it affects their daily life and requires medical attention. Further research is now required to further elucidate the epidemiology of this occupational issue, including identifying key risk factors and their impact on employment, so that appropriately targeted interventions may be instigated.

## Abbreviations

MSD: Musculoskeletal disorders

## Competing interests

The authors declare that they have no competing interests.

## Authors’ contributions

All authors were involved in the study design, data analysis and manuscript preparation, and approved the final manuscript.
